# What to Survey? A Systematic Review of the Choice of Biological Groups in Assessing Ecological Impacts of Metals in Running Waters

**DOI:** 10.1002/etc.4810

**Published:** 2020-08-11

**Authors:** Hiroki Namba, Yuichi Iwasaki, Jani Heino, Hiroyuki Matsuda

**Affiliations:** ^1^ Graduate School of Environment and Information Sciences Yokohama National University Yokohama Kanagawa Japan; ^2^ Nippon Koei Tokyo Japan; ^3^ Research Institute of Science for Safety and Sustainability National Institute of Advanced Industrial Science and Technology Tsukuba Ibaraki Japan; ^4^ Freshwater Center, Finnish Environment Institute Oulu Finland; ^5^ Faculty of Environment and Information Sciences Yokohama National University Yokohama Kanagawa Japan

**Keywords:** Ecological risk assessment, Acid mine drainage, Mine effluents, Aquatic insects, Diatoms, Fish

## Abstract

Which biological groups (in the present study, periphyton, macroinvertebrates, and fishes) are surveyed is a fundamental question in environmental impact assessment programs in metal‐contaminated rivers. We performed a systematic review of 202 studies that investigated the ecological impacts of metal contamination on aquatic populations and communities in streams and rivers to examine 1) which biological groups were surveyed, 2) whether their responses were correlated with each other, and 3) which biological group was most responsive to changes in metal contamination level. In these studies, published from 1991 to 2015, benthic macroinvertebrates were most frequently chosen throughout the period (59–76% in different 5‐yr periods), followed by periphyton and fishes, and the number of studies that surveyed at least 2 or 3 biological groups was very limited (10%). Pearson's correlation coefficients calculated between the metrics of different biological groups were often low, emphasizing the importance of investigating multiple biological groups to better understand the responses of aquatic communities to metal contamination in running waters. Despite the limited data collected, our meta‐analysis showed that, in most cases, biological metrics based on macroinvertebrates were more responsive to changes in metal contamination level than those based on periphyton or fishes. This finding suggests that benthic macroinvertebrates could be a reasonable choice to detect the ecological impacts of metal contamination on a local scale. *Environ Toxicol Chem* 2020;39:1964–1972. © 2020 The Authors. *Environmental Toxicology and Chemistry* published by Wiley Periodicals LLC on behalf of SETAC.

## Graphical abstract

Which biological groups (periphyton, macroinvertebrates, or fishes) are surveyed is a fundamental question in environmental impact assessment programs in metal‐contaminated rivers.

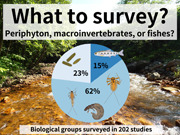

## INTRODUCTION

Freshwater ecosystems harbor high levels of biodiversity and support important ecosystem services that humans rely on, yet these ecosystems are currently being modified and stressed by various anthropogenic impacts (Flitcroft et al. 2019; Reid et al. 2019). For example, ecological impacts of trace metal contamination in freshwaters caused by mining activities, including legacy mines, are a long‐standing concern worldwide (Luoma and Rainbow 2008; Iwasaki and Ormerod 2012; Giam et al. 2018). Ecological risk assessments can provide vital information on how the ecological risks of trace metal contamination should be managed. Although comparing environmental metal concentrations with environmental water or sediment quality benchmarks is useful for screening‐level ecological risk assessments (Chapman 2018; van Dam et al. 2019), biological assessments based on field surveys of natural populations and communities are imperative for understanding the ecological consequences of exposure (Barbour et al. 1999; Crane et al. 2007; Schmidt et al. 2010; Iwasaki et al. 2011; Iwasaki and Ormerod 2012; Peters et al. 2014).

For biological assessments in rivers and streams, the typical biological groups used are periphyton, benthic macroinvertebrates, and fishes (Barbour et al. 1999; Birk et al. 2012). These individual groups have different advantages in bioassessment programs (Barbour et al. 1999). Briefly, periphyton primarily consists of algae that are primary producers supporting riverine food webs; they have short life cycles (which potentially makes them a valuable indicator of short‐term physical and chemical impacts), are easy to sample, and are often highly sensitive to anthropogenic disturbances (Barbour et al. 1999; Biggs 2000). Macroinvertebrates are relatively sedentary, have variable life cycles ranging from many generations per year to one generation in several years, are easy to sample, and comprise diverse sets of species with a wide range of sensitivities to trace metals (Barbour et al. 1999; Rosenberg et al. 2008; Iwasaki et al. 2018). These characteristics indicate that macroinvertebrates are useful for assessing site‐specific impacts and for observing relatively local‐scale and long‐term cumulative effects (Barbour et al. 1999). In general, fishes are relatively long‐lived and mobile, their life histories and distributions are comparatively well known, and so they are good indicators of longer term and broad‐scale effects (Barbour et al. 1999). Also, fishes are important sources of food for humans and are used for recreational and commercial fishing, and they thus generally have a higher value than other groups for many local human communities.

Which biological groups are surveyed in environmental assessment programs in metal‐contaminated streams and rivers is a fundamental question before biological assessments are initiated, and the choice depends on the management goals agreed on among various stakeholders. Information about which biological groups are most commonly used in evaluating ecological impacts of trace metals in streams and rivers, and also information about cross‐taxon congruence among the different biological groups (i.e., correlation among responses of multiple biological groups; Heino 2010), would be useful to inform assessment plans. A few studies (e.g., Heino 2010; de Morais et al. 2018) summarized findings about the utility of cross‐taxon congruence in aquatic ecosystems and concluded that no single taxonomic group can be used to reasonably predict responses of other groups to the environment. However, even if the cross‐taxon congruence is low, it is useful to know which biological group is most responsive to metal contamination; then we could monitor the group that would respond before the ecological impacts of management concern occur. However, all these pieces of information are largely lacking in the assessment of ecological impacts of metals in running waters.

In the present study, we focused on studies that investigated the ecological impacts of metal contamination on aquatic populations and communities in streams and rivers. We carried out a systematic review to examine 1) which biological groups (i.e., periphyton, macroinvertebrates, or fishes) were surveyed; 2) whether their responses correlated (i.e., the degree of among‐taxon congruence); and 3) which biological group was most responsive to changes in the metal contamination level. We did not include studies that investigated accumulation of trace metals in aquatic organisms (i.e., periphyton, macroinvertebrates, and/or fishes) but did not address the impacts of metal contamination on aquatic populations and communities.

## MATERIALS AND METHODS

### Data collection

We searched relevant peer‐reviewed journal articles (hereafter termed case studies) published between 1991 and 2015 in the Web of Science core collection database on 30 June 2019. We used the following terms for the search: metal* AND (stream* OR river*) AND (bryophyte* OR alga* OR diatom* OR periphyton* OR macroinvertebrate* OR invertebrate* OR benthos* OR “aquatic insect*” OR fish*) AND (population* OR communit* OR assemblage* OR abundance* OR densit* OR richness OR diversit* OR biomass OR metric* OR index* OR indices) AND document type: (Article). The operator “*” is a wildcard to match one or more words.

The document type was set to “Article” to exclude publications other than original research articles (e.g., reviews). For this search, “density,” “abundance,” “richness,” “biomass,” and “index” were included as biological metrics to assess population‐ or community‐level effects. The word “index” was included to incorporate studies using biological indices such as the multimetric indices of biotic integrity (Karr 1981; Barbour et al. 1999).

A total of 1506 studies were found. We then sorted out relevant studies based on the ecological effects investigated, type of studied system, studied factors of concern, studied organisms, and sampling methods. We first excluded studies that did not investigate the effects of metals on aquatic populations or communities in rivers, as judged from the title, abstract, and/or the body text. Typical excluded studies were those investigating only metal contamination in environmental media, such as surface water and river sediment, those examining only the accumulation of metals in biological species, those conducted in habitats other than freshwater rivers (i.e., lakes, bays, and estuaries), and those investigating impacts of anthropogenic factors other than metals (e.g., land use and organic chemical compounds).

We included studies analyzing at least 1 of the 4 trace metals (zinc [Zn], copper [Cu], cadmium [Cd], and lead [Pb]) in river water, discharged from point sources (mainly legacy and active metal mines), and also included studies performed in rivers affected by coal mines because it was often impossible to discriminate metal from coal mines from the available information. These selection criteria excluded only a few studies performed in specific mines such as arsenic [As] and molybdenum [Mo] mines. In addition to the 4 trace metals, nickel [Ni] and As were analyzed in approximately 20% of studies finally examined, and iron [Fe], aluminum [Al], and manganese [Mn], together with pH, were often analyzed in the studies performed in coal mines. In terms of pH, studies conducted in both near‐neutral and acidic rivers were included. We then excluded studies whose major objective was not to examine the impacts of metals but to investigate the effects of anthropogenic factors in general (e.g., water quality in urban rivers).

We defined benthic macroinvertebrates as benthic communities typically dominated by aquatic insects, and studies focusing only on crustaceans or mollusks were excluded. This was simply because commonly employed sampling methods do not aim to collect particular taxonomic groups but rather a mix of benthic communities collected from certain areas (Barbour et al. 1999; Friberg et al. 2006; Birk et al. 2012). Finally, we excluded studies other than those directly based on data from natural communities (i.e., studies using colonization tiles or trays were excluded), because sampling methods and periods varied among such studies and, therefore, it was difficult to determine whether the results reflected natural effects.

On the basis of the sampled organisms, we classified the remaining individual studies into the following 7 categories: 1) periphyton, 2) macroinvertebrates, 3) fishes, 4) periphyton and macroinvertebrates, 5) periphyton and fishes, 6) macroinvertebrates and fishes, and 7) periphyton, macroinvertebrates, and fishes. To answer the first question (which biological groups are surveyed?), we counted the number of studies sampling periphyton, macroinvertebrates, or fishes and evaluated the selection frequency of each biological group. Studies sampling more than one biological group were counted more than once. In addition, we evaluated the numbers of studies that sampled a single biological group and 2 or 3 biological groups, and only the latter studies were used for subsequent analysis (see the following section, *Correlation of changes among biological groups*).

### Correlation of changes among biological groups

To quantitatively examine the correlations between different pairs of biological groups, we collected raw data (i.e., values of biological metrics), generally directly from original tables or from original figures, by using ImageJ software (National Institutes of Health 2019). Because of the limited availability of studies and data (see the *Results* section for more details), only 10 pairs (comparisons) from 8 studies were included in the present analysis. Those studies fell into 2 types. Studies of the first type were based on samplings at a single or a few time points at multiple sites within a river or multiple rivers, giving more attention to spatial than temporal variations in metal contamination and its ecological effects. Studies of the second type were based on temporally repeated samplings at multiple sites, aiming to investigate temporal changes in metal contamination and the corresponding biological changes (typically, recovery). Thus, for the correlation analyses, we divided the studies into 2 categories, “spatial variation” and “temporal change.”

For each study, by checking the interpretations and conclusions of the original articles, we first evaluated whether any effect was detected for each biological group sampled. Although this evaluation was qualitative, such information was useful for interpreting the calculated correlation coefficients. For instance, if an effect of metal contamination was not observed for both of the biological groups examined, the correlations between biological metrics could be low.

To test the magnitude of cross‐taxon congruence, we calculated Pearson's correlation coefficients (*r*) between biological metrics (i.e., abundance [number of individuals], density, richness [number of taxa], diversity, biomass, and/or multimetric index) for different biological groups. Abundance, density, and biomass were log_10_‐transformed for the present analysis. If multiple metrics were available for individual biological groups in the original studies, the correlation coefficients were calculated in all combinations. The range (minimum and maximum) of *r* and the number of high positive correlations (operationally defined as *r* > 0.7), as well as the number of all combinations, were reported for each pairwise comparison within a study. For the temporal change studies, we calculated the correlation coefficients at each sampling site separately, because the relationships between 2 biological groups often depended on focal sites (see the subsequent paragraph for more details). If one of the 2 metrics was expected to increase in response to metal contamination (i.e., percentage of dominant taxon for periphyton [Pool 2013] and percentage of chironomids [Carline and Jobsis 1993]), the calculated correlation coefficients were multiplied by −1.

As an additional analysis to better understand factors affecting the magnitudes of the correlations in the temporal change studies, we tested the hypothesis that higher correlations between different biological groups (i.e., stronger cross‐taxon congruence) are observed at sites that experienced more severe metal contamination. This is expected because the improvement of water quality after severe contamination would likely cause significant changes in any biological group. We quantitatively tested this hypothesis by examining the relationships between site‐median values of correlation coefficients and the levels of metal contamination, although only 2 temporal change studies had sufficient data for this analysis.

### Responses of biological groups to metal contamination

To investigate which biological group was more responsive to changes in metal contamination, we calculated Pearson's correlation coefficients between the levels of metal contamination and biological metrics (listed in the previous section, *Correlation of changes among biological groups*) for the 3 biological groups in individual studies. As indicators of metal contamination, we collected values of the indicators reported in the original studies depending on the major concerns (i.e., the total Zn or dissolved Cu concentration, the cumulative criterion unit [CCU], or pH). The CCU was used as a measure of exposure to metal mixtures in a study and was calculated as the ratio of the dissolved concentrations of 3 metals to the hardness‐adjusted US Environmental Protection Agency water quality criteria (Cd, Cu, and Zn for Clements [2010] and Cd, Pb, and Zn for Maret and MacCoy [2002]; Maret et al. 2003). Of the 10 comparisons, raw data on biological metrics from 8 comparisons, from which raw data on the indicators of metal contamination could also be obtained, were analyzed.

## RESULTS

**Figure 1:**
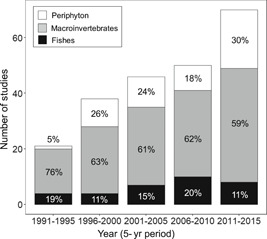
Temporal changes in the number of studies published between 1991 and 2015 that surveyed periphyton, macroinvertebrates, or fishes to assess the ecological impacts of metal contamination in rivers. Percentages of the number of corresponding studies in each period are shown.

On the basis of our review of 1506 articles, we collected 202 studies that investigated the ecological impacts of metal contamination on aquatic populations and communities in streams and rivers. Most studies were performed in North America and Europe (49 and 28%, respectively), but studies conducted in Oceania (10%), Asia (9%), South America (3%), and Africa (1%) were also available (see Supplemental Data for more details). Periphyton was surveyed in 52 studies, macroinvertebrates were surveyed in 140 studies, and fishes in 33 studies. The total numbers of studies increased from 1991 to 2015, and benthic macroinvertebrates were most frequently chosen throughout the period (59–76%), followed by periphyton and fishes (Figure 1). These percentages were almost constant among different continents (i.e., North America, Europe, Oceania, and Asia; see Supplemental Data for more details). Most studies (90%; *n* = 181) sampled only a single biological group, and the number of studies that surveyed 2 or 3 biological groups was very limited (10%; *n* = 20). The number of studies surveying 2 or 3 biological groups ranged from 2 to 6 in individual 5‐yr periods (1991–2015), and no clear temporal trend was observed (see Supplemental Data for more details).

Of these 20 studies, we could obtain the raw data to calculate correlation coefficients only for 9 comparisons from 7 studies. For this analysis, we also used information from 2 studies that were published in 2 different journals but surveyed macroinvertebrates and fishes at identical sites in the same sampling periods (Maret and MacCoy 2002; Maret et al. 2003; hereafter, these combined field results were counted as a single study). Among them, 5 were spatial variation studies and 3 were temporal change studies (Table 1). All except one study were performed in the United States. In spatial variation studies, all of the pairwise combinations of periphyton, macroinvertebrates, and fishes were available, whereas only the combination of macroinvertebrates and fishes was available in temporal change studies (Table 1). In total, the numbers of biological metrics compiled for periphyton, macroinvertebrates, and fishes were 8, 24, and 11, respectively. For periphyton and macroinvertebrates, 13% of the metrics were indices of biotic integrity, and richness/diversity and abundance/biomass metrics almost equally split the remaining 87% (see Supplemental Data for more details). For fishes, 80% represented abundance/biomass metrics dominated by trout species, and the remaining were indices of biotic integrity.

**Table 1:** Summary information of the “spatial variation” and “temporal change” studies surveying multiple biological groups in metal‐contaminated rivers

Category	ID	Group	No. of metrics			No. of data points^a^	Range of *r* values	Median *r* values^b^	Strong correlation ratio^c^	Reference
			P	M	F					
Spatial variation									
	1	P + M	1	1	NA	8	−0.42	−0.42	0/1	De Jonge et al. (2008)
	2	P + M	1	4	NA	6	−0.17–0.57	−0.10	0/4	Bott et al. (2012)
	3	P + M	6	1	NA	13	0.25–0.76	0.60	1/6	Pool et al. (2013)
	4	P + F	6	NA	1	8	−0.05–0.63	0.32	0/6	Pool et al. (2013)
	5	M + F	NA	4	4	4	−0.83–0.93	−0.16	3/16	Carline and Jobsis (1993)
	6	M + F	NA	1	1	7	0.87	0.87	1/1	Pool et al. (2013)
	7	M + F	NA	5	2	18	−0.54–0.45	0.03	0/10	Maret and MacCoy (2002); Maret et al. (2003)
Temporal change									
	8	M + F	NA	1	1	4	0.00–0.88	0.00–0.88	1/2	Underwood et al. (2014)
	9	M + F	NA	4	2	10–11	0.03–0.84	0.28–0.80	8/32	Clements et al. (2010)
	10	M + F	NA	4	1	8–11	−0.68–0.78	−0.38–0.74	4/24	Mebane et al. (2015)

^a^ Number of data points corresponds to the numbers of sites in spatial variation studies and to the numbers of observations in temporal change studies.

^b^ The ranges of median *r* values (Pearson's correlation coefficients) estimated based on multiple study sites are shown for temporal change studies.

^c^ Strong correlation is defined as a Pearson's correlation coefficient value of >0.7.

P = periphyton; M = macroinvertebrates; F = fishes; NA = not applicable.

**Figure 2:**
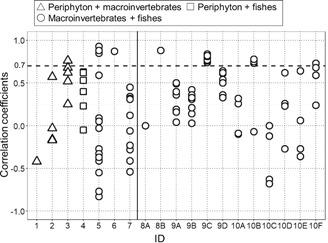
Pearson's correlation coefficients between metrics based on different biological groups in spatial variation (ID 1–7) and temporal change studies (ID 8–10; see Table 1 for more details). Letters show the site numbers in individual studies (see Supplemental Data for more details). The horizontal dashed line indicates a selected threshold correlation coefficient of 0.7, denoting a strong correlation.

**Figure 3:**
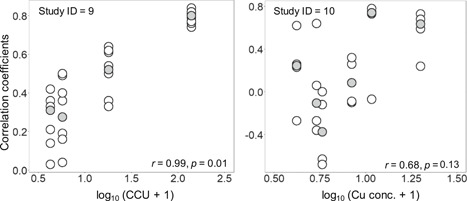
Relationships between Pearson's correlation coefficients of the metrics based on macroinvertebrates and fishes and metal contamination levels in 2 studies (ID 9 and 10; see Table 1). Metal contamination levels were defined as the highest cumulative criterion unit of metals (CCU) values or the highest Cu concentrations (µg/L) observed at individual sites during the study periods. Gray circles are medians of correlation coefficient (*r*) values at each site. The *r* and corresponding *p* values shown in the panels were calculated from site‐median values. The metrics used for these correlation analyses were abundance/biomass and richness of macroinvertebrates and abundance/biomass of 2 trout species (brown trout for ID 9; rainbow trout for ID 10).

Only one spatial variation study found no effects of metal contamination on macroinvertebrates and fishes, with *r* values varying widely (median: −0.27, range: −0.83–0.93; Table 1 [ID = 5] and Figure 2). In other spatial variation studies and in temporal change studies, which concluded that effects were observed for both biological groups, median *r* values also varied largely (−0.42–0.88). The percentages of high correlation (*r* > 0.7) between different biological groups were 11% (5/44 combinations) in spatial variation studies and 22% (13/58 combinations) in temporal change studies (Figure 2). Of the 18 correlation coefficients with *r* > 0.7, all except one periphyton–macroinvertebrates combination were the combinations between macroinvertebrates (mostly, their richness or abundance) and fishes (mostly, trout abundance). Note that the combinations including periphyton were relatively limited in our dataset (17/102 combinations). In addition, the median values of correlation coefficients and the contamination levels were positively correlated in the 2 temporal change studies with at least 4 study sites (*r* = 0.99 and 0.68), although the correlation was not statistically significant in one of the studies (Figure 3).

**Figure 4:**
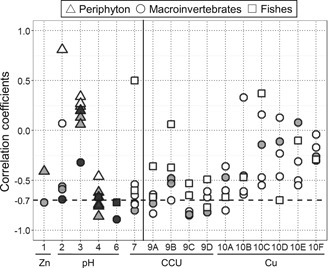
Pearson's correlation coefficients between biological metrics and the indicators of metal contamination in “spatial variation” (ID 1–7; numbers in *x*‐axis) and “temporal change” studies (ID 9 and 10; see Table 1 for more details). The indicators of metal contamination were Zn concentration (ID 1), pH (ID 2–6), cumulative criterion unit (CCU; ID 7 and 9), and Cu concentration (ID 10). Letters show the site numbers in individual studies (see Supplemental Data for more details). Symbols filled with white, gray, and black are abundance/biomass metric, richness/diversity metric, and multimetric index of biotic integrity, respectively. The horizontal dashed line indicates a correlation coefficient of −0.7. For studies that used pH as an indicator of metal contamination, the correlation coefficients were multiplied by −1.

In only one spatial variation study (ID 3, 4, and 6 in Figure 4), all 3 biological groups (periphyton, macroinvertebrates, and fishes) were surveyed. In that study, the biological metrics based on macroinvertebrates had higher correlations with pH than those of periphyton and fishes, although the sampling sites were not identical among the 3 comparisons (see Supplemental Data for more details). Three other spatial variation studies also showed that the metrics based on macroinvertebrates were more highly correlated with the metal contamination level than those based on periphyton. In 90% of the cases in the temporal change set of studies (i.e., except for 10D in Figure 4), the richness or abundance metrics for macroinvertebrates were more strongly correlated with changes in metal contamination than trout abundance or biomass.

## DISCUSSION

### Choice of biological groups surveyed

Our systematic literature review showed that benthic macroinvertebrates have been most frequently chosen in the assessment of ecological impacts of metals on populations and communities in running waters, whereas periphyton and fishes have been surveyed at rates of ≤30%. This result is generally consistent with the results of Davis et al. (1996) and Birk et al. (2012). Birk et al. (2012) showed that benthic invertebrates were the most dominant biological group used in 297 biological assessment methods in 28 European countries. Although elucidating the reasons underlying the selection frequency is difficult, the advantages of using benthic macroinvertebrates (detailed in the *Introduction*) and the historical background of using macroinvertebrates to assess the impacts of organic contamination in running waters (e.g., saprobic systems; Kolkwitz and Marsson [1909]) should have played an important role, as was discussed by Birk et al. (2012). It should be emphasized that, because we did not include studies examining only metal accumulation in organisms, the inclusion of such studies would have likely changed the selection frequency of different biological groups (particularly, that of fishes) used in biological assessments.

The number of studies that surveyed at least 2 or 3 biological groups was limited (10%) in the set of studies we found. This is rather unexpected despite the relatively long history of encouraging or requiring biological monitoring of at least 2 biological groups in the United States (Davis et al. 1996; Barbour et al. 1999), Canada (Environment Canada 2012), and Europe (European Commission 2000). For instance, the metal mining environmental effect monitoring program initiated by the 2002 Metal Mining Effluent Regulations in Canada requires both fish and benthic invertebrate monitoring (Walker et al. 2003). Although probably not substantial, some results of biomonitoring of individual biological groups were found to be published in different journals, leading to a difficulty in matching those results. For example, Maret and MacCoy (2002) and Maret et al. (2003) are from the same study but are published in a fish journal and an invertebrate journal, respectively. The most likely reason is that, although many relevant monitoring datasets have been collected, few of them have been published in peer‐reviewed scientific journals. Publishing those monitoring studies as well as making use of those relevant datasets would be critically important to perform both a more comprehensive meta‐analysis and more detailed analyses on the cross‐taxon congruence and relative sensitivity of biological groups to metal contamination.

### Cross‐taxon congruence and sensitivity of biological groups

The correlations between the changes in different biological groups (i.e., periphyton, macroinvertebrates, and fishes) were often low (Figure 2). Also, if the “strong” correlation is defined as *r* > 0.9 instead of the *r* > 0.7 used in the present study, the percentages decrease from 11 to 2% in the spatial variation studies and from 22 to 0% in the temporal change studies. However, the results of our further analysis indicated positive correlations between correlation coefficients and the levels of metal contamination, although the available data were again very limited (i.e., only 2 studies; Figure 3). Similar results were obtained for the relationships between correlation coefficients and the magnitudes of changes in metal contamination levels (Supplemental Data, Figure S1). These results provide modest support to our hypothesis that stronger cross‐taxon congruence can be observed at sites that have experienced more severe metal contamination and have subsequently shown improved water quality.

The weak cross‐taxon congruence may be explained largely by the apparent differences in the sensitivities of the 3 biological groups to metal contamination. Indeed, our meta‐analysis showed that the biological metrics based on macroinvertebrates (mostly richness and abundance) were more responsive to changes in metal contamination than those based on periphyton or fishes in most comparisons (Figure 4). Similarly, some case studies concluded that fish metrics, such as density or multimetric index, were less sensitive to the metal contamination than macroinvertebrate metrics (Freund and Petty 2007; Clements et al. 2010). Furthermore, although this factor is not metal contamination, fishes are generally less responsive to specific conductivity, a measurement of concentration of ions such as Ca^2+^ and Mg^2+^, than macroinvertebrates, based on extirpation concentrations estimated from field sampling at >3000 stations in the central and southern Appalachians (USA; Griffith 2017; Griffith et al. 2018). As was detailed in the *Introduction* section, such different sensitivities that we observed should be partly associated with differences in the spatial–temporal scales of organisms' responses to the environment (Barbour et al. 1999; Heino et al. 2005; Freund and Petty 2007; Infante et al. 2009).

Because observed sensitivities of biological groups to metal contamination depend on how the responses were measured and the kinds of biological metrics collected were limited (particularly for periphyton and fishes) in the present study, caution is required for interpretation of our findings. For example, for periphyton, changes in species composition and diversity are more sensitive indicators of metal contamination than changes in biomass or primary production rate (Hirst et al. 2002; Niyogi et al. 2002; De Jonge et al. 2008). For macroinvertebrates, taxon richness measures such as total taxa richness and mayfly taxa richness are generally more reliable indicators than abundance and ratio measures in terms of sensitivity, variability, and statistical power (Carlisle and Clements 1999), although the abundances of specific taxa, such as heptageniid mayflies, can be highly responsive to changes in the metal contamination level (Clements et al. 2000; Schmidt et al. 2010; Iwasaki et al. 2018). In addition, although trout abundance metrics dominated among the fish metrics collected in the present study, small‐bodied sedentary fish species, such as sculpins, may be more sensitive to metal contamination than relatively large‐sized and potentially mobile fish species (Gibbons et al. 1998; Maret and MacCoy 2002). Also, even though several studies concluded that the multimetric indexes of biotic integrity can be used to detect the impacts of metal contamination (Mebane 2003; Bervoets et al. 2005), the component metrics may be more responsive. The inherent issues and limitations of using such indexes have also been discussed before (Suter 1993). Finally, although population‐level and community‐level effects were addressed among the 3 biological groups in the present study, physiological and biochemical metrics, such as fish body condition, can be more sensitive to detect the population‐level consequences (Munkittrick and Dixon 1989; Hanson 2009; Environment and Climate Change Canada 2015). Therefore, understanding and considering how the responses are measured should be important in guiding rigorous testing of cross‐taxon congruence and in selecting the most sensitive biological groups in assessing the ecological impacts of metal contamination on aquatic biota. However, it has to be noted that the limited data availability hindered more detailed analyses in the present study.

The correlations between the levels of metal contamination and biological metrics may have varied depending on the exposure indicators. In this regard, although we simply used exposure indicators of concern in the original studies, our conclusions were not fundamentally affected if other exposure indicators reported were employed for the correlation analysis (e.g., Cd or As instead of Zn in ID 1, and Fe instead of pH in ID 2, 3, 4, and 6). Nevertheless, biological metrics observed in the fields are likely influenced by other abiotic and biotic factors, which may have affected our results. For example, metal toxicity can be complicated by water chemistry affecting metal bioavailability (Adams et al. 2020) and the combination of trace metals (Meyer et al. 2015). Also, the metal contamination in sediments, particularly if it is not correlated with the contamination in water, may have played an important role in appropriately interpreting the results. Overall, these issues highlight the long‐standing need for increasing our understanding of ecological impacts and its causes at individual study sites or in individual rivers. Accumulation of such knowledge would help guide the choice of biological groups for assessing the ecological impacts of metal contamination.

This systematic review demonstrated that the correlations among the responses of periphyton, macroinvertebrates, and fishes in metal‐contaminated rivers were generally low, emphasizing the importance of investigating multiple biological groups to better understand the responses of aquatic communities to metals (Freund and Petty 2007; Heino 2010; de Morais et al. 2018). Our results also suggest that, among the 3 biological groups, benthic macroinvertebrates may be the indicator group most responsive to metal contamination. Despite the caveats pointed out above, in environmental impact assessments without any specific preference for the aquatic organisms investigated and without sufficient budget for surveying multiple biological groups, benthic macroinvertebrates could be a reasonable first choice to detect the ecological impacts of metal contamination on a local scale. More detailed studies, including case studies from different types of aquatic systems, would be required to test the generality of our findings on the selection of biological groups to examine the effects of metal contamination in running waters.

## Supplemental Data

The Supplemental Data are available on the Wiley Online Library at https://doi.org/10.1002/etc.4810.

## Author Contributions Statement

Y. Iwasaki and H. Namba conceived and designed the study inspired by J. Heino. H. Namba and Y. Iwasaki performed the literature review and data analysis. H. Namba, Y. Iwasaki, J. Heino, and H. Matsuda wrote and revised the manuscript.

## Supporting information

This article includes online‐only Supplemental Data.

Supporting information.

Supporting information.

## Data Availability

Data and associated metadata are available in the Supplemental Data, and interested parties should contact the corresponding author (yuichiwsk@gmail.com) for further details including calculation tools.
