# Supplementary material for: What to Survey? A Systematic Review of the Choice of Biological Groups in Assessing Ecological Impacts of Metals in Running Waters
Source: Environ Toxicol Chem. 2020 Aug 11;39(10):1964–72. doi: 10.1002/etc.4810 (PMC7590085; doi:10.1002/etc.4810)
Supplement: Supplementary file 2 — Supporting information. [file ETC-39-1964-s002.docx]

**Supplemental Data**

Figure S1. Relationships between correlation coefficients of the metrics based on macroinvertebrates and fishes and metal contamination levels in two studies (ID 9 and 10; see Table 1). The magnitudes of changes in metal contamination were defined as the range (maximum − minimum) of CCU (cumulative criterion unit of metals) values or that of Cu concentrations (µg/L) observed at individual sites during the study periods. Gray circles are medians of Pearson’s correlation coefficients (*r*) at each site. The *r* and corresponding *p* values shown in the panels were calculated from site-median values.
